# A Retrospective Investigation of 28 Cats with Intermediate- to Large-Cell Lymphoma Treated with Lomustine and Prednisolone as a First-Line Chemotherapy

**DOI:** 10.3390/ani16060989

**Published:** 2026-03-22

**Authors:** Hee-Je Kim, Rayoung Heo, Eun Wha Choi

**Affiliations:** 1 Department of Veterinary Clinical Pathology, College of Veterinary Medicine & Institute of Veterinary Science, Kangwon National University, 1 Kangwondaehak-gil, Chuncheon 24341, Republic of Korea; 2 N Animal Hospital Nowon, 408 Nowon-ro, Nowon-gu, Seoul 01704, Republic of Korea

**Keywords:** feline lymphoma, intermediate- to large-cell lymphoma, lomustine, survival rate, progression-free interval, treatment response

## Abstract

Lymphoma is the most common neoplastic disease in cats; however, lomustine has been insufficiently evaluated as a first-line chemotherapeutic agent. This study assessed treatment response, survival, and progression-free interval (PFI) in cats with intermediate-to-large cell lymphoma treated with lomustine and prednisolone as first-line therapy. Twenty-eight cats with cytologically or histopathologically confirmed lymphoma received lomustine (10 mg/cat every 3 weeks) and prednisolone until disease progression or unacceptable toxicity. All cats were feline leukemia virus- and feline immunodeficiency virus-negative, with a mean age of 9.93 years; alimentary lymphoma was most common (75%). Treatment responses included complete response (CR, 5/28), partial response (5/28), stable disease (11/28), and progressive disease (7/28). The median PFI for all cats was 51 days, and the median survival time was not reached during the study period. No significant associations were identified between PFI or survival and age, sex, packed cell volume, drug dosage, tumor size, or tumor location. Cats achieving CR showed significantly prolonged PFI compared with cats with other responses (median, 561 vs. 42 days; *p* = 0.0004), and overall survival was also significantly longer (*p* = 0.0009). These findings may serve as a clinically meaningful guide for the use of lomustine and prednisolone in the treatment of feline lymphoma.

## Simple Summary

Lymphoma is one of the most common cancers in cats, and choosing an effective treatment that is also practical for owners is an important challenge in veterinary medicine. Lomustine is an oral anticancer drug that can be given at home, but its use as an initial treatment for feline lymphoma has not been well studied. In this study, we evaluated how cats with intermediate- to large-cell lymphoma responded to treatment with lomustine combined with prednisolone. Twenty-eight cats were treated and monitored for treatment response, length of survival, and time until the disease worsened. Most cats showed either temporary improvement or stabilization of their disease, and a small number achieved complete disappearance of detectable cancer. Cats that achieved complete response lived significantly longer and had a longer period without disease progression than cats with other responses. These results suggest that lomustine combined with prednisolone may be a useful first treatment option for selected cats with intermediate- to large-cell lymphoma and may help veterinarians and owners make informed treatment decisions.

## 1. Introduction

Lomustine, also known as 1-(2-Chloroethyl)-3-cyclohexyl-1-nitrosourea (CCNU), is an orally administered alkylating agent with active metabolites that exert cytotoxic effects in vivo [1]. This property supports its use as a chemotherapeutic option in feline lymphoma. A previous study described the in vivo metabolism of lomustine into its active metabolites in dogs, which showed a metabolic profile comparable to that of humans, with trans-4-hydroxylomustine being the major metabolite [2]. To the best of our knowledge, no detailed pharmacokinetic studies of lomustine have been previously reported in cats; therefore, most of the available information has been extrapolated from dogs or general veterinary pharmacology references, rather than from feline-specific data. Owing to its lipid solubility and low molecular weight, lomustine can effectively cross the blood–brain barrier [3,4,5,6].

Lymphoma is the most common type of tumor in cats [7,8]. According to a study involving cats receiving veterinary care at VetCompass-participating clinics in the United Kingdom, the prevalence of lymphoma was 0.048% (271/562,446) [9]. Lymphoma is also associated with feline leukemia virus (FeLV) infection, with most FeLV-related lymphomas arising from T-cell lineages and primarily manifesting as mediastinal or peripheral forms [10]. However, the alimentary form is typically FeLV-negative [8]. Gastrointestinal (GI) lymphoma is the most common form of lymphoma in cats, primarily affecting those aged 10–12 years, compared to those aged 4–6 years for FeLV-related lymphoma [11]. Lymphomas associated with feline immunodeficiency virus (FIV) are generally of B-cell origin, tend to occur in extranodal locations, and typically affect older cats [12].

Lymphoma in cats can be classified into small- and intermediate- to-large-cell types based on the cell size, which differ in terms of clinical presentation, biological behavior, and therapeutic response. Small-cell lymphomas often present as chronic indolent diseases, are frequently alimentary, and respond well to treatment with chlorambucil and prednisolone. In contrast, intermediate- to large-cell lymphomas are more aggressive, commonly involve multiple anatomical sites, and require multiagent chemotherapy to ensure optimal management [11]. Generally, the treatment protocol for intermediate- to-large-cell lymphomas in cats involves cyclophosphamide, vincristine, prednisolone or prednisone (COP) or cyclophosphamide, doxorubicin, vincristine, prednisolone or prednisone (CHOP). CHOP protocols are associated with nephrotoxicity and higher costs and may require frequent visits and prolonged hospitalization [13,14], which can be burdensome for both feline patients and their guardians. In contrast, COP protocols are less nephrotoxic and do not require long hospital stays or weekly visits after the initial four-week induction period, thereby reducing treatment-related stress [15]. Lomustine-based protocols also offer advantages, such as convenient oral administration and minimal hospitalization, making lomustine a practical and less stressful first-line chemotherapeutic option for feline lymphoma.

A few retrospective studies have examined the responses of feline lymphoma patients to single-agent lomustine treatment [4,5,6]. However, Dutelle et al. did not use lomustine as the first-line chemotherapy agent [4]. Fan et al. focused on various types of tumors, with only six lymphoma cases included in their study [5]. In a study by Rau et al., lomustine and prednisolone were used as first-line therapies; however, some cats underwent surgery, and others received L-asparaginase [6].

In particular, this study aimed to assess the survival and PFI in cats with intermediate- to large-cell lymphoma treated exclusively with lomustine and prednisolone by excluding those that underwent surgery or other rescue treatments such as CHOP-based protocols. In clinical veterinary oncology practice, lomustine is rarely administered as a true single agent without concurrent corticosteroids [16]. Prednisolone is commonly included because of its well-recognized lympholytic effects and its ability to provide rapid clinical improvement and palliation in cats with lymphoma [4,6,17]. Accordingly, the treatment protocol used in the present study was designed to reflect real-world clinical practice rather than to evaluate lomustine monotherapy. The potential prognostic factors analyzed for associations with survival time and PFI included age, sex, packed cell volume (PCV), lomustine dose relative to body surface area, tumor size, and tumor location.

## 2. Materials and Methods

### 2.1. Patient Selection and Inclusion Criteria

This retrospective study was approved by the Kangwon National University Institutional Animal Care and Use Committee (approval number: KW-240326-1). The data used in this study were obtained from the medical records of the N Animal Hospital Nowon (Seoul, South Korea) between January 2021 and December 2025. Cats diagnosed with intermediate- to large-cell lymphoma and treated with lomustine (10 mg per cat) and prednisolone (0.5–1 mg/kg twice daily or 2 mg/kg once daily) as first-line chemotherapy on a continuous every-three-week schedule were included in the study.

### 2.2. Exclusion Criteria

Cats that underwent surgery or received other treatment protocols such as subsequent CHOP-based protocols were excluded from the analysis.

### 2.3. Diagnostics

Tumor sizes in cats were measured using various imaging modalities (ultrasonography, X-ray, or CT), and diagnoses of intermediate- to large-cell lymphoma were made based on cytological (n = 27) or histopathological examinations (n = 1). All cats included in this study were tested for FeLV and FIV using an IDEXX SNAP kit (SNAP Feline Triple Test, IDEXX, Westbrook, ME, USA).

### 2.4. Treatment

Cats were treated with lomustine (10 mg per cat) and prednisolone (0.5–1 mg/kg twice daily or 2 mg/kg once daily). Lomustine was administered continuously at 3-week intervals until progressive disease (PD) or unacceptable toxicity occurred. Treatment was withheld if the neutrophil count was ≤3000/μL or the platelet count was ≤100,000/μL. For cats receiving only one or two doses of lomustine, if a treatment response or symptomatic improvement was observed, prednisolone was tapered by 50% every two weeks until discontinuation, with patients monitored every three weeks. After three or more doses of lomustine, if a treatment response or symptomatic improvement was observed, prednisolone was tapered by 50% every two weeks until discontinuation, with patients monitored every three weeks. In addition, prednisolone was discontinued immediately in patients who developed elevated fructosamine levels.

After disease progression, treatment consisted of prednisolone, as the owners declined more aggressive treatments such as CHOP or COP. Supportive care, including antiemetics, appetite stimulants, and GI protectants, was provided as needed based on the patient’s clinical condition. Additional interventions, such as application of a buprenorphine transdermal patch (or fentanyl patch) or placement of a nasoesophageal tube (or esophagostomy tube), were performed when indicated, and euthanasia was undertaken when clinically appropriate and at the owner’s request.

### 2.5. Treatment Response Assessment

Tumor assessments were performed at three-week intervals. Tumor measurements were performed using imaging, and when objective measurements were not possible, the response was assessed based on clinical signs. Tumor response categories were defined according to the World Health Organization criteria outlined in a previous study [18]: Complete response (CR) was defined as complete disappearance of the tumor for at least 21 days, A partial response (PR) was defined as >50% but <100% reduction in size of all measurable disease for 21 days. Stable disease (SD) was defined as <50% reduction in measurable disease for 21 days with no new lesions arising during that time period. PD was defined as >25% increase in measurable disease or the appearance of new lesions. Transient decreases in measurable disease that persisted for <21 days were defined as PD.

### 2.6. Evaluation of Survival Time and PFI

The survival time, PFI, and treatment response were evaluated in 28 cats. The analysis of PFI and survival time was based on previous studies. Briefly, PFI was defined as the time from the start of lomustine treatment until disease progression or euthanasia [6]. Although PFI generally includes the time until a change in treatment, cats that underwent a change in treatment were not included in our study; therefore, this component was excluded from the PFI definition. For the PFI, feline patients whose tumors had not progressed were censored at the date of their last visit. For the survival time, feline patients who were alive, died from another cause, or were lost to follow-up were censored at the date of their last visit. All cats classified as having PD owing to a lack of response to lomustine and prednisolone were included to minimize any potential bias in the survival analysis and PFI.

### 2.7. Toxicity Assessment

During lomustine treatment, complete blood counts (CBC) were measured every three weeks using an automated hematology analyzer (ADVIA 2120i, Siemens, Erlangen, Germany).

### 2.8. Prognostic Factors for PFI and Duration of Survival

The potential prognostic factors analyzed included age, sex, anemia, drug dosage, tumor size, and tumor location. Sex (male vs. female), and tumor location (non-GI vs. GI) were treated as categorical variables, whereas the remaining factors were analyzed as continuous variables.

### 2.9. Statistical Analysis

Survival time and PFI were estimated using Kaplan–Meier curves. Univariate Cox regression analyses were performed to evaluate the association between each prognostic factor and PFI or overall survival. Hazard ratios with 95% confidence intervals were calculated for each factor. Survival curves were estimated using the Kaplan–Meier method, and differences between groups were assessed using the log-rank test. Statistical significance was set at *p* < 0.05. All analyses were performed using GraphPad Prism software (version 10.02, GraphPad Software, Boston, MA, USA) or SPSS software (version 29, IBM Corp., Armonk, NY, USA).

## 3. Results

### 3.1. Feline Patient Characteristics

Details of the feline patient characteristics, including breed, sex, age, weight, cell size, tumor location, and FeLV and FIV test results, are summarized in Table 1.

The mean age of the cats was 9.93 years (SD: 2.64), and the mean weight was 4.74 kg (SD: 1.68). The most common breed was Korean Short Hair, followed by Persian, Russian Blue, and Scottish Folds.

The tumors were located in the stomach (n = 15), small intestine (n = 5), colon (n = 1), intra-abdominal multicentric lymph nodes (n = 3), mediastinum (n = 1), spleen (n = 1), kidney (n = 1), and nasal cavity (n = 1). Lymphomas arising in the stomach, small intestine, or colon were classified as GI lymphomas (n = 21), whereas those in other locations were classified as non-GI lymphomas (n = 7). All 28 cats, regardless of their body surface area, were treated with lomustine (10 mg/cat) every three weeks. A 40 mg lomustine capsule was divided and re-encapsulated to yield 10 mg doses, which were subsequently used. The median lomustine dose administered was 35.7 mg/m^2^ (range: 22.5–66.1 mg/m^2^). Lomustine was administered from one to a maximum of 17 times (median: 3 times), with distribution as follows: one time (n = 4), two times (n = 4), three times (n = 9), four times (n = 1), five times (n = 4), six times (n =1), eight times (n = 2), 10 times (n = 1), 11 times (n = 1), and 17 times (n = 1).

### 3.2. Tumor Assessment and Imaging Results

Treatment response at all anatomical sites (n = 26), except the nasal cavity and mediastinum, was evaluated using ultrasonography, with tumor size reduction indicating treatment effectiveness. Three weeks after the initial lomustine administration, ultrasound examinations were performed in all 26 cats, except for those with nasal cavity or mediastinal lymphoma. After this time point, three cats died, while the remaining 21 cats underwent ultrasound monitoring at three-week intervals, and two cats were monitored intermittently. The cat with mediastinal lymphoma underwent thoracic radiography and the cat with nasal lymphoma underwent head CT and radiography. Ultrasonographic findings suggestive of tumor presence include nephromegaly with a halo sign in cases of renal lymphoma, segmental wall thickening (maximum central thickness, 2.1 cm), loss of normal layering, and hypoechogenicity in cases of GI lymphoma [19]. Fine-needle aspiration (FNA) was performed under ultrasound guidance when these findings were observed, and cytology confirmed the diagnosis of lymphoma. For GI tumors in which FNA was challenging, endoscopic biopsy and histopathology were used for a definitive diagnosis.

In the cat with nasal lymphoma (n = 1), tumor size was assessed based on the presence or absence of clinical signs owing to the impracticality of repeated CT under anesthesia. In the cat with mediastinal lymphoma (n = 1), tumor size was evaluated using radiography. Tumor response in cases of renal lymphoma was monitored via ultrasonography to assess serial changes in size and evaluate potential invasion into adjacent organs and serum chemistry parameters related to renal function.

### 3.3. Responses to Treatment

Chemotherapy was well tolerated in all cats, with no therapy-associated deaths or intensive care requirements. Neutropenia (neutrophil count ≤ 3000/μL) occurred in six of the 28 cats during the treatment period. According to the VCOG-CTCAE v2 criteria for evaluating hematologic toxicity [20], six cats were classified as grade 1 (1500/μL–< lower limit of normal). In cats with leukopenia, G-CSF (Leucostim Injection 150 mcg, Dong-A ST Co., Ltd., Seoul, South Korea) was administered subcutaneously at 10 μg/kg, followed by CBC re-evaluation after 1–2 weeks; lomustine was re-administered after recovery from leukopenia.

Based on the patient’s clinical condition and CBC results, lomustine was administered continuously at intervals of 3–8 weeks. In cases where CR was maintained for more than one year, lomustine treatment was discontinued, and cats were subsequently monitored at monthly intervals (n = 2).

One cat with nasal lymphoma received six doses of lomustine. Although the size of the nasal mass appeared to decrease based on gross assessment of visible swelling, clinical signs worsened after the sixth administration, and the cat ultimately died. Accordingly, the lesion was classified as SD.

The treatment responses of the 28 cats were as follows: CR, 5/28; PR, 5/28; SD, 11/28; and PD, 7/28. A summary of the treatment responses is presented in Table 2.

### 3.4. PFI and Survival Times

The PFI for all cats was 51 days (range, 14–569 days; censored cases ≥ 569 days, Table 2). The median survival time (MST) for all cats was not reached during the study period; observed survival times ranged from 23 to 671 days; censored cases ≥ 671 days (Table 2).

**Figure 1:**
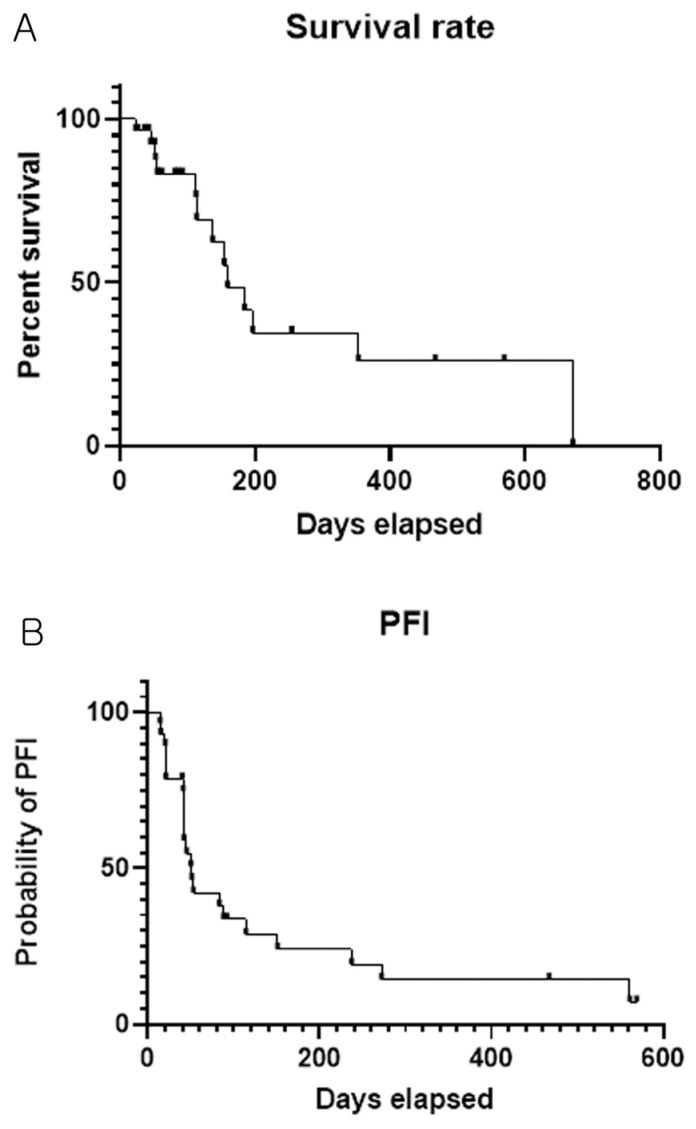
(**A**) Kaplan–Meier survival curve illustrating the probability of survival for 28 cats treated with lomustine and prednisolone. The MST for the 28 cats was not reached with observed survival times ranging from 23 to 671 days. (**B**) Kaplan–Meier curve illustrating the PFI for 28 cats treated with lomustine and prednisolone. Median PFI for the 28 cats was 51 days (range: 14–569 days). The Log-rank test was used to analyze survival rate data.

Kaplan–Meier survival curves illustrating the probability of survival for the 28 cats treated with lomustine and prednisolone, as well as the PFI for the 28 cats treated with the same regimen, are presented in Figure 1A,B.

### 3.5. Prognostic Factors and Consequences of the Outcome

The results of the analyses of PFI and survival time for the six parameters are presented in Table 3. When analyzed according to age, sex, PCV, drug dosage, tumor size, and tumor location, none of the factors were found to be predictive of PFI or overall survival. As no parameters were significant in univariate analysis, multivariate Cox regression analysis was not performed.

**Figure 2:**
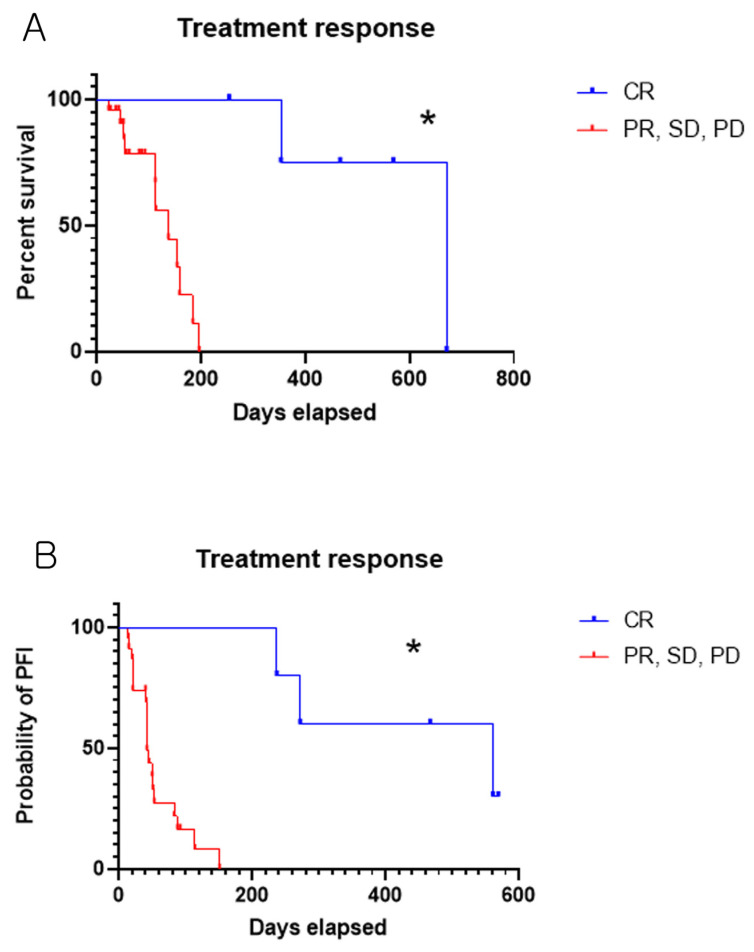
(**A**) Kaplan–Meier survival curve was generated based on the treatment responses (CR versus PR + SD + PD). (**B**) Kaplan–Meier curve of PFI based on the treatment responses (CR versus PR + SD + PD). The Log-rank test was used to analyze survival and PFI data, with *p* < 0.05 (*) considered statistically significant. PFI: Progression-free interval, CR: Complete response, PR: Partial response, SD: Stable disease.

Cats that received three or fewer doses of lomustine (n = 17) had an MPFI of 42 days (range, 14–561 days), whereas those that received four or more doses (n = 11) had an MPFI of 151 days (range, 21–569 days; censored cases ≥ 569 days). This difference was statistically significant (log-rank test, *p* = 0.0047). Cats treated with lomustine three or fewer times had a 2.860-fold higher risk of shorter PFI than those who received more than three doses (hazard ratio based on log-rank analysis). Lomustine was administered continuously at 3-week intervals until PD or unacceptable toxicity occurred. In most cats, lomustine treatment was continued until disease progression or death, which was the primary reason for discontinuation. Therefore, the number of doses received largely reflected the clinical response of individual cats rather than serving as an independent factor for predicting the outcome. This suggests that cats with a more favorable response to lomustine experienced extended disease control and survival, allowing more doses to be administered. The MPFI for the CR cases was 561 days (range, 237–569 days; censored cases ≥ 569 days), whereas that for the other response cases (PR + SD + PD) was 42 days (range, 14–151 days), showing a statistically significant difference (log-rank test, *p* = 0.0004). By contrast, the MST was not reached in either group; however, cats achieving CR showed significantly prolonged overall survival compared with cats with other responses (PR + SD + PD) (log-rank test, *p* = 0.0009). Observed survival times ranged from 353 to 671 days (censored cases ≥ 671 days) in the CR group and from 23 to 196 days in the other response group. Kaplan–Meier survival and PFI curves based on treatment response are presented in Figure 2A and 2B, respectively.

## 4. Discussion

This study evaluated the overall response of cats diagnosed with intermediate- to large-cell lymphomas to lomustine and prednisolone as first-line anticancer drugs. As many owners in this study preferred to avoid frequent hospital visits and intensive treatment protocols, cats were evaluated and treated with lomustine at three-week intervals, following the protocol described by Fan et al. [5]. According to Fan et al., owing to biohazard concerns associated with the reformulation of lomustine capsules, all cats were administered a single 10 mg capsule every 21 days regardless of body weight. In their study, up to 12 doses were administered and severe hematological toxicity was rarely reported. In the Republic of Korea, lomustine 10 mg capsules are not commercially available. As a safety measure, lomustine from 40 mg capsules was re-encapsulated into 10 mg lomustine using gelatin capsules inside a biosafety cabinet while wearing a mask and gloves. For quality control, 40 mg of lomustine was re-encapsulated to 10 mg using an analytical balance, and the weight was subsequently verified using the analytical balance after re-encapsulation.

In the present study, the age distribution of affected cats followed a normal distribution, with a mean age of 9.93 years and a median age of 11 years. This is considerably higher than the median age of 4–6 years reported in previous studies conducted before the introduction of the FeLV vaccine [21,22]. In previous studies, the proportion of FeLV-positive cats was high (approximately 70%), and mediastinal lymphoma was the most commonly reported subtype. In contrast, all 28 cats in our study tested negative for FeLV, and the alimentary form was predominant, accounting for 75.0% of the cases, whereas mediastinal lymphoma was observed in only 3.6% of cats. Similarly, in a study conducted in the United States, 25.5% of cats were FeLV-positive, the median age of the affected cats was 9.5 years, and alimentary lymphoma was the most frequent subtype (50%), followed by multicenter (27%) and mediastinal lymphoma (17%) [23].

In this study, cats achieving CR demonstrated prolonged disease control and survival. The median PFI (MPFI) was 561 days, whereas the MST was not reached at the time of analysis. Two of the five cats in the CR group had died at 353 and 671 days after treatment initiation, while the remaining three cats were still alive with ongoing follow-up at 254, 467, and ≥569 days. These findings indicate sustained clinical benefit in cats achieving CR, although longer follow-up is required to fully characterize long-term survival outcomes.

Interpretation of survival outcomes in this study is limited by a substantial early-censoring pattern. Most censored cases occurred during the early follow-up period, whereas death events were relatively sparse and tended to occur later. This distribution of events may have precluded estimation of the MST using Kaplan–Meier analysis. For reference, among cats with confirmed death events, the median post-PFI survival time (i.e., survival after the onset of PD) was 59 days (range, 3–138 days). Potential prognostic factors, including age, sex, PCV, lomustine dose relative to body surface area, tumor size, and tumor location, were analyzed for associations with survival time and PFI; however, no statistically significant associations were observed in this study. However, given the small number of cases, these results should be interpreted with caution, and it would be premature to conclude that these factors have no clinical relevance.

Survival times for cats with untreated lymphoma are generally very short, although not all studies have categorized feline patients based on lymphoma site. In two early studies, the average survival time for untreated or supportively treated cats ranged from 5 days (16 cats) to 2 weeks (15 cats) [24,25].

The COP protocol is commonly used as a first-line treatment for feline lymphoma. The reported outcomes vary across studies, depending on whether the results are presented for the entire treated population or only for feline patients achieving CR.

In one study reporting outcomes specifically for feline patients achieving CR, the COP protocol yielded a CR rate of 36% and a MPFI and MST of 1139 days [15]. In another study of 61 cats treated with COP, the CR rate was 75.4% and the median duration of response and MST in feline patients who achieved CR were 251 and 266 days, respectively. In contrast, cats that failed to achieve CR had a minimal likelihood of surviving beyond one year [26]. Studies reporting outcomes for the overall treated population found substantially shorter survival times; in 114 cats treated with COP, the MPFI and MST were 65.5 and 108 days, respectively [27].

For CHOP-based protocols, including the modified 25-week University of Wisconsin–Madison regimen, the reported MPFI and MST for the overall treated population were 56 days and 97 days, respectively [28].

Although these studies differ in terms of case distribution, treatment intensity, and whether outcomes are reported for the overall population or only for feline patients achieving CR, the collective findings consistently indicate that cats that achieve CR have substantially longer PFI and survival than those that do not.

While a direct comparison is not feasible owing to differences in the study populations and follow-up durations, the CR rate observed in cats treated with lomustine and prednisolone (5/28, 17.9%) was lower than that reported for the COP and CHOP protocols in the literature. Nevertheless, the MST and MPFI were similar across the treatment groups. Moreover, consistent with previous studies, feline patients who achieved CR had significantly longer PFI and survival than cats that did not achieve CR. The lower CR rate observed with lomustine and prednisolone compared with COP or CHOP protocols is likely due to the less intensive nature of this regimen [16]. COP and CHOP combine multiple agents with different mechanisms of action, achieving more rapid and profound tumor cytoreduction. Lomustine, administered as a single cytotoxic drug with prednisolone, may have slower or less complete antitumor effects, particularly in aggressive lymphoma subtypes. Despite the lower CR rate, cats achieving CR still demonstrated prolonged progression-free and overall survival, highlighting the clinical utility of this convenient and less intensive protocol.

As mentioned previously, CHOP protocols are associated with nephrotoxicity, higher costs, and the need for frequent hospital visits and prolonged stays, which may pose a significant burden to cats and their owners. In contrast, COP protocols carry a lower risk of nephrotoxicity and typically do not require weekly visits after the initial four-week induction period or extended hospitalization, thereby minimizing treatment-related stress. Lomustine-based regimens provide similar benefits with the added convenience of oral administration and minimal need for hospitalization, making lomustine a practical and less stressful first-line chemotherapeutic option for feline lymphoma [15]. Therefore, lomustine and prednisolone were chosen as anticancer agents in this study, as they are less stressful for both the cat and the owner, while still achieving a certain degree of efficacy. Cats can experience stress from oral medication administration; however, most cats do not experience stress that is severe enough to interfere with their daily activities. In this study, a few cats exhibited mild stress related to the oral administration of lomustine, such as transient drooling; however, no cats experienced severe stress causing anorexia, difficulty in urinating, or disruption of daily activities.

The limitations of this study include the small sample size (28 cats), which may limit the statistical power and generalizability of the findings. Tumors were broadly categorized as GI or non-GI due to the limited number of non-GI cases, potentially obscuring location-specific outcomes. Weekly hematologic and biochemical monitoring was not consistently performed, as most owners preferred three-week interval visits, which may have led to missed transient hematologic nadirs or biochemical changes. Additionally, immunophenotypic classification of lymphoma cells into T-cell or B-cell lineages was not performed, precluding analysis of lineage-specific treatment responses. Future studies with larger cohorts, systematic monitoring, and immunophenotyping are warranted to better evaluate prognostic factors and treatment outcomes in feline lymphoma.

Despite these limitations, this study provides valuable insights into the treatment response, survival, and PFI associated with the use of lomustine and prednisolone as first-line chemotherapy for feline lymphoma. These findings may serve as a useful clinical reference for the application of lomustine in the management of feline lymphoma.

## 5. Conclusions

In this study, we evaluated MPFI and MST in cats with intermediate- to-large cell lymphoma treated with lomustine and prednisolone as first-line chemotherapy. Among the 28 cats, treatment responses were CR in 5/28, PR in 5/28, SD in 11/28, and PD in 7/28. The MPFI for all cats was 51 days (range, 14–569 days; censored cases ≥ 569 days), whereas the MST was not reached, with observed survival times ranging from 23 to 671 days (censored cases ≥ 671 days). Cats achieving CR had a significantly longer PFI than those with other responses (median, 561 vs. 42 days), and survival was significantly prolonged in cats achieving CR compared with cats showing other responses, as demonstrated by Kaplan–Meier analysis and the log-rank test.

## Figures and Tables

**Table 1:** Characteristics of the 28 cats included in this study.

Variable	Category	Mean ± SD	n	%
Breed	Korean short hair		14	50.0
	Persian		4	14.3
	Russian blue		3	10.7
	Scottish fold		2	7.1
	Siamese		1	3.6
	Mix		1	3.6
	American shorthair		1	3.6
	British shorthair		1	3.6
	Turkish angora		1	3.6
Gender	Castrated male		11	39.3
	Spayed female		16	57.1
	Female		1	3.6
Age	0–2 years		0	0.0
	3–4 years		1	3.6
	5–6 years		3	10.7
	7–8 years	9.93 ± 2.64 years	5	17.9
	9–10 years		4	14.3
	11–12 years		11	39.3
	13–14 years		4	14.3
Weight		4.74 ± 1.68 kg		
Cell size	Intermediate		14	50.0
	Large		14	50.0
Location	Alimentary		21	75.0
	Multicentric		1	3.6
	Mediastinal		3	10.7
	Spleen		1	3.6
	Kidney		1	3.6
	Nasal		1	3.6
FeLV test FIV test	Negative Negative		28 28	100.0 100.0

SD: Standard deviation, FeLV: Feline leukemia virus, FIV: Feline immunodeficiency virus.

**Table 2:** Treatment response, PFI and survival outcomes for cats receiving lomustine.

Response	n/N (%, 95% Confidence Intervals)
CR	5/28 (17.9%, 7.9–35.6%)
PR	5/28 (17.9%, 7.9–35.6%)
SD	11/28 (39.3%, 21.5–59.4%)
PD	7/28 (25.0%, 10.7–44.9%)
Median duration of response (CR + PR)	237 (42–569) days
Median PFI (CR + PR + SD)	83 (41–569) days
Median PFI (CR + PR + SD + PD)	51 (14–569) days
**Median survival time**	**Median (min–max)**
Overall (CR + PR + SD + PD)	Not reached
Responders and stable (CR + PR + SD)	184 (45–671) days
Responders (CR + PR)	353 (45–671) days

CR: Complete response, PR: Partial response, SD: Stable disease, PD: Progression disease, PFI: Progression-free interval.

**Table 3:** Factors predicting PFI and survival.

Patient Characteristic	PFI (n = 28)		Survival (n = 28)	
	*p*	Hazard Ratio (95% CI)	*p*	Hazard Ratio (95% CI)
Age	0.782	1.021 (0.879–1.186)	0.510	1.071 (0.874–1.312)
Sex (Male vs. Female)	0.637	1.231 (0.519–2.921)	0.436	1.601 (0.490–5.228)
PCV	0.833	0.993 (0.926–1.064)	0.340	1.054 (0.946–1.176)
Drug dosage	0.746	1.010 (0.952–1.071)	0.830	0.990 (0.905–1.083)
Size of tumor	0.700	0.992 (0.954–1.032)	0.059	0.928 (0.858–1.003)
Location of tumor (non-GI vs. GI)	0.257	1.788 (0.654–4.889)	0.712	0.802 (0.250–2.576)

Univariate Cox regression analyses were performed to evaluate the associations between each prognostic factor and PFI or overall survival. Hazard ratios with 95% confidence intervals were calculated for each factor. Statistical significance was set at *p* < 0.05. PFI: Progression-free interval, CI: Confidence interval, PCV: packed cell volume, GI: Gastrointestinal.

## Data Availability

The data used in this study are available from the corresponding author upon request.

## Abbreviations

The following abbreviations are used in this manuscript:

CCNU	1-(2-Chloroethyl)-3-cyclohexyl-1-nitrosourea
FeLV	Feline leukemia virus
FIV	Feline immunodeficiency virus
GI	Gastrointestinal
PFI	Progression-free intervals
CBC	Complete blood count
CR	Complete response
PR	Partial response
SD	Stable disease
PD	Progressive disease
MST	Median survival time
PCV	Packed cell volume
MPFI	Median progression-free interval
